# Type 2 diabetes in a rapidly urbanizing region of Ghana, West Africa: a qualitative study of dietary preferences, knowledge and practices

**DOI:** 10.1186/1471-2458-14-1069

**Published:** 2014-10-14

**Authors:** Megan L Doherty, Ellis Owusu-Dabo, Osei Sarfo Kantanka, Rickie O Brawer, James D Plumb

**Affiliations:** School of Population Health, Thomas Jefferson University, Philadelphia, PA USA; Global Health Programs Office, Perelman School of Medicine, University of Pennsylvania, Philadelphia, PA USA; Kumasi Center for Collaborative Research in Tropical Medicine, Kwame Nkrumah University of Science and Technology, Kumasi, Ghana; Diabetes Clinic, Komfo Anokye Teaching Hospital, Kumasi, Ghana; Center for Urban Health, Thomas Jefferson University, Philadelphia, PA USA; Department of Family and Community Medicine, Jefferson Medical College, Thomas Jefferson University, Philadelphia, PA USA

## Abstract

**Background:**

Urban centers in Sub-Saharan Africa, such as Kumasi, Ghana, are especially impacted by the dual burden of infectious and non-communicable disease (NCD), including a rise in type 2 diabetes mellitus (T2DM) prevalence. To develop effective intervention programs, the World Health Organization recommends more research to better understand the relationship between food consumption and the escalation of non-communicable disease such as T2DM. This study provides qualitative information about current food knowledge, attitudes and practices among T2DM patients and their caregivers in the region of Kumasi, Ghana.

**Methods:**

In this qualitative study, three focus groups discussions of 30 persons total and 10 individual interviews were used to assess food preferences, knowledge, attitudes and practices of patients with T2DM as well as caregivers responsible for food preparation. Participants included both urban and rural dwellers. Hospital-based health talks were observed, a dietician was interviewed, and educational documents were collected. Themes were identified and coded using Nvivo10 software.

**Results:**

Findings suggest that messages regarding sweetened foods, fats, use of seasonings and meal timing are followed. However, confusion exists regarding the impact of fruits, food portioning, plantains and processed foods on health outcomes for diabetic patients. Results also revealed a problem-solving approach to increasing vegetable consumption, and a concern about unhealthy food preferences among younger generations.

**Conclusions:**

Education about the impact of commonly available carbohydrates on blood sugar should be emphasized; messaging on portion sizes and certain foods should be more consistent; the economic benefits of local vegetable consumption should be promoted; and a research-informed, T2DM prevention campaign should be developed specifically for younger generations.

## Background

Type 2 diabetes mellitus (T2DM) ranks as one of the most rapidly increasing non-communicable diseases (NCDs) in the world today. Over 371 million people worldwide were diagnosed with diabetes in 2011, with an expected 7.7% increase by 2030 [1]. This increase is especially pronounced in urban Sub-Saharan Africa (SSA) where soaring diabetes rates are expected to double within that same timeframe [1]. In fact, the World Health Organization (WHO) projects that NCDs, such as T2DM, will overtake infectious, maternal, perinatal and nutritional diseases as the leading cause of mortality on the African continent by 2030 [2]. Despite these projections, only 1% of global healthcare spending is directed towards diabetes in Africa as international funding agencies, research centers and health systems are still primarily focused on the region’s infectious disease load [3].

Urban populations, in regions such as SSA, are considered to be at especially high risk for diabetes due to the nutritional shift from a minimally-processed, rural diet to a more ‘Westernized’ diet quickly gaining popularity in cities [4–6].

Several recent studies recommend more research on nutritional factors related to the global rise of T2DM to improve efficacy of intervention programs [7–9]. For instance, the WHO’s 2003 report on Diet, Nutrition and the Prevention of Chronic Disease concluded that it is “crucial to obtain more reliable information on actual food consumption patterns, diets and trends based on representative consumption surveys” in order to understand the relationship between food consumption and the escalation of NCDs [9]. The purpose of this study is to provide qualitative information about current food knowledge, attitudes and practices among T2DM patients and their caregivers in a rapidly urbanizing region of SSA.

### Research setting

Ghana, West Africa, has been significantly affected by a dual burden of infectious disease and NCDs, including a rise in diabetes prevalence [10, 11]. Specifically, the prevalence of Ghanaian adults with diabetes is expected to double from 500,000 in 2010 to nearly one million by 2030 [12]. Kumasi, the capital of the Ashanti region, is Ghana’s second largest city and has a higher rate of urbanization than the capital, Accra [13]. According to the Ghana Health Service, diabetes rates have been steadily increasing in the Kumasi region, registering as the 10^th^ leading cause of death from 2004–2006 [14]. Further, two recent studies conducted in urban Kumasi, and a surrounding area, found that diabetes rates for these populations were 2-4% higher than the average among urban, adult West Africans [15, 16].

At the national level, Ghana’s Ministry of Health (MOH) is aware of the threat to national public health posed by T2DM. Consequently, the MOH’s National Diabetes Management and Research Centre is currently in the process of enhancing the country’s national diabetes program through a partnership with the World Diabetes Foundation (WDF). The new program, developed between October 2009 and September 2013, is slated to include “a culturally appropriate and specific curriculum and program instruments for the control and prevention of diabetes in Ghana” [17]. Planned relevant innovations include a surveillance system to monitor risk factors including “unhealthy eating” and population-based prevention activities implemented in churches, mosques and other community gathering places [17].

## Methods

**Figure 1:**
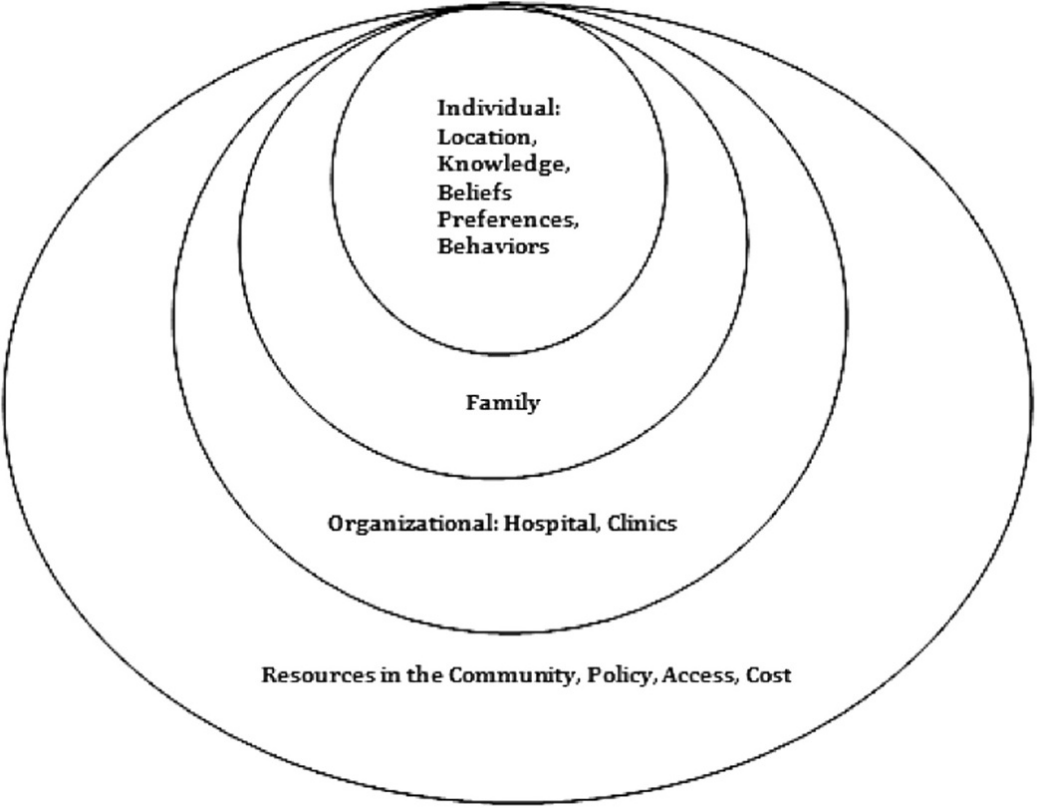
**Ecological behavioural model.**

This qualitative study adhered to guidelines for relevancy, appropriateness, transparency and soundness in qualitative research [18]. A fixed set of ‘a priori’ interview questions were based on the ecological model (Figure 1) which postulates that certain behaviors may be influenced by the individual’s context such as: urban or rural dwelling; personal preferences; impact on family; involvement with the hospital diabetes clinic; and access to fresh foods, to name a few [19].

Interview questions centered on variables influencing health outcomes such as: nutritional knowledge; dietary preferences; dietary beliefs; dietary behavior; disease management; information channels; and barriers and enabling factors.

Focus group discussions (FGDs) included only people receiving care for diabetes; semi-structured individual interviews were conducted with caregivers of such individuals. Two separate, but similar, survey instruments were used for these two groups: a FGD guide and an individual interview guide. Behavior-related questions in the FGD guide were aimed at understanding the individual’s food intake and general disease management whereas similar questions in the interview guide centered on the caregiver’s food shopping and preparation practices. Additionally, caregivers were asked, broadly, about changes within the household resulting from the family member’s diabetes diagnosis. Otherwise, knowledge and attitudes regarding food choice related to diabetes were the same. The Ghanaian authors of this study approved the final FGD and individual interview questions as culturally appropriate and relevant.

A mix of Kumasi city and rural residents was interviewed in both the FGDs and individual interviews; for the purposes of this study, ‘rural’ was defined as ≥40 km outside of Kumasi.

The Thomas Jefferson University Institutional Review Board and the Kwame Nkrumah University of Science and Technology Committee on Human Research Publication and Ethics approved this study.

Focus group participants were recruited through convenience sampling in the waiting area of the diabetes clinic at Komfo Anokye Teaching Hospital (KATH). A clinic nurse introduced the researcher and research assistant to the persons in the waiting area; a Ghanaian research assistant explained the goals of the study in the predominant regional language (Twi) and requested volunteers. Three focus groups of ten persons per group were conducted during two diabetes clinic days. All participants met the inclusion criteria of ≥18 years of age and with a diagnosis of diabetes. Each focus group included males and females as well as diverse age groups, educational levels and residential statuses. To recruit individual interviewees, the study used snowball sampling from those FGD participants reliant on caregivers to prepare their meals (often, males and the female elder in a multi-generation household do not prepare their own meals in Ghana). All individual interviewees also volunteered to take part in the study. For caregivers, semi-structured, individual interviews in their homes or workplaces were conducted. Demographic survey questions included the diabetes patient’s gender, age, number of years since diagnosis, residential status (urban/rural), and highest level of education obtained. Individual interviews of caregivers included the above demographic questions for the patient, as well as the gender, age and highest educational level of the caregiver.

To protect identity, each participant was assigned a unique identifier that was used in the place of names on demographic survey and interview instruments. Results of both instruments were coded to the numbers assigned to the participants. At the request of the participants, the FGDs and all but two of the interviews were conducted in Twi and voice recorded. For those conducted in Twi, the research assistant translated all FGDs and interviews into English while a researcher simultaneously transcribed them. Afterwards, the research assistant cross-checked the written notes with the Twi voice recordings for fidelity. At the close of each session, all participants were provided with small ”gifts” such as cell phone airtime, apples or avocados.

Observation, document review, and further discussion were used to substantiate the information obtained from the focus groups and interviews. Specifically, a health talk provided in the diabetes clinic waiting room was transcribed. Additionally, a dietician at KATH was interviewed on the hospital’s approach to educating diabetic patients about diet, and copies of menu planning handouts were obtained. Themes among all data were identified and independently coded using NVivo10 software. Themes were reviewed and agreed upon during a coding conference, and an outside researcher reviewed the themes for congruency.

## Results

### Characteristics of sample

Of the 30 participants diagnosed with diabetes, 10 (33%) were male and 20 (66%) were female. The mean ± standard deviation age of males was 54.1 ± 8.9 and 48.8 ± 13.4 for females. For both males and females, the mean number of years since diagnosis was 9 with ±6.9 and 6.3 respectively. All of the 10 caregivers interviewed were female with mean ± SD age 48.7 ± 10.

Most (57%) participants with diabetes resided in Kumasi, while 43% were rural dwellers. Of the caregivers, the number was divided evenly between urban and rural residents.

A range of educational levels was represented among the participants. Most (55%) of the interviewees had attained Junior High School-level schooling. Seven (17.5%) participants reported to have had no formal schooling at all, and four (10%) completed primary school only. Two completed up to a high school degree, two a technical or commercial high school and three completed university studies. One participant had attained a master’s degree.

### Responses

#### Dietician interview and observation of health talks at KATH

Patients with diabetes at KATH receive individualized counseling and educational handouts during the hospital’s diabetes clinic days. Dieticians interview patients with diabetes to understand current eating habits including preferences, portion sizes and eating schedules. If there are clear problem areas, the dietician negotiates with the patient in an effort to correct it by instituting a diet plan. The diet plan is aimed at weight management, as well as nutrition, and includes: general eating guidelines; foods to avoid; and recommendations on foods and quantity for all three meals and two snacks. Dietary recommendations in the case of illness and low blood sugar are also included. At the end of the counseling session, patients are provided all of this information in the form of an English language, text-only handout.

In addition to the dietician’s counseling, outreach workers provide health talks in the KATH Diabetes Clinic waiting area where patients gather in the morning for the day’s appointments. These talks reinforce information provided by the dietician as well as other important messages regarding common dietary misconceptions (e.g., “plantains cure diabetes”), the importance of proper foot care; and support services available for people with diabetes such as KATH’s Diabetes Patient Association.

### Dietary preferences

#### Home cooked meals

There was a strong preference for home-cooked meals among both urban and rural dwellers throughout all FGD and individual interviews. Eating outside of the home was actually seen as undesirable. Food preparation time was not a hindrance for the vast majority of participants. In fact, all participants in one focus group echoed this statement made by one participant:

“There is nothing better than homemade meals. When it takes a long time we eat something small to sustain us until it’s ready. Time is not a factor because we prefer to cook and eat homemade.”

Porridge (millet or maize) was the most commonly reported breakfast choice and often included bread or an egg, though two participants reported only having tea and bread. Three people from rural areas, as well as one person in the urban area, reported eating pounded yam or plantain with a combination of leaf, vegetable and fish stews for breakfast. All participants reported eating or serving home-cooked meals for lunch and dinner, which included a staple starch or grain with a soup or stew, and sometimes supplemented with bread. The preferred staple starches were yam, cocoyam, cassava, and plantain; staple grains were millet, maize and rice. Soups and stews most often included those with main ingredients of palm oil, cocoyam (taro) leaves, tomatoes, onions, meats, and fresh, salted, smoked or dried fish.

#### Local foods vs. packaged foods

Local, home cooked foods were preferred over packaged foods by all participants (Though some packaged foods are produced in Ghana, participants often referred to these same foods as ‘imported’). Most participants simply described locally grown and freshly prepared foods as “healthier” than packaged foods. Some participants gave more specific reasons, including:

“Local foods are healthier than packaged because of the sugar content. If you want to live long, avoid packaged foods” (46-year-old urban male);

“Local food is good and healthy. Packaged food is not healthy because of the ingredients....what’s on the label may not be true” (55-year-old rural female); and

“You can’t compare them because the imported has chemicals. But our food, when you prepare it and you prepare it well, that one is excellent.” (62-year-old urban female).

In contrast, participants felt that younger people preferred sugar-sweetened beverages, ‘sweets’, highly seasoned street-foods (grilled meat, fried rice, meat pies, etc.) and beer to the local, home-cooked foods. A 60-year-old urban female even suggested an unhealthy consequence to this preference:

“Older people eat differently. Young people prefer sweet things. Also, younger people prefer lots of meat. They can sit at a beer bar and order lots of meat and before you know it, they have a potbelly. Young people don’t really have worries. They live their lives however they want.”

### Knowledge and practices

#### Added sugar and sweet-tasting foods

Both rural and urban dwellers frequently acknowledged the importance of avoiding added sugar in managing diabetes in the FGDs, as well as in each of the individual interviews with the caregivers. Discussion mostly centered on omitting sugar in porridges, avoiding sugar-sweetened beverages (including juices), as well as processed foods, in general, because of high sugar content. Fruit, on the other hand, was generally not considered to be detrimental to a diabetes management plan. In fact, one 60-year-old, urban female recommended that people with diabetes “should eat more oranges, banana, pineapple, and apples”. A few participants mentioned that fruit intake should be limited because of high sugar content, though most did not make a link between fruits and sugar. In fact, fruit was a commonly reported snack among the participants; the most commonly named fruits were bananas, mangoes, watermelon, oranges, avocados and apples.

In one case, a participant pointed out that she has Milo (pre-sweetened chocolate drink) daily but does not add sugar to it. Many also specifically mentioned eating bread but avoiding “sugar bread”. There were also several mentions of eating only the biscuits and crackers that “do not contain sugar”. Several of the diabetic participants did mention using soft drinks (especially Coca Cola and Malta) as a source of energy when feeling tired, when no food is available, or in response to feeling shaky or faint.

#### Plantains

Plantains were one of the most commonly reported starches eaten or served. Discussion about plantains ranged from a belief that they are recommended for diabetes management to an understanding that they are unhealthy for people with diabetes. The following quotes illustrate the variety of beliefs among participants concerning plantains:

“People should eat more plantain and less food that contains carbohydrates like cassava.....I learned this from a nurse” (55-year-old rural female)

“Previously we were told to eat a lot of plantain and not the other starchy foods so the older generation held on strongly to these views. These days we are told that we can eat almost everything, but rather add a lot of soup or stew.....I too attended a private clinic some time ago, the doctor did not tell me to avoid plantain but I heard it from other patients....Some people come and tell our women’s groups that plantain is good for diabetics. I don’t say anything but then I tell the other diabetic patients later that it’s not true.” (59-year-old rural female)

#### Bread, biscuits and crackers

Eating bread was mentioned in all of the FGDs and in all but three of the individual interviews. As mentioned above, many participants clarified that they eat or serve the kind of bread “without sugar”. Eating or serving bread for breakfast, whether with tea alone, or with porridge and tea, was commonly reported. Bread was also eaten or served at lunchtime as a supplement to the meal; as just as a snack when feeling hungry; and sometimes with a piece of fruit, such as mango or avocado.

Biscuits and crackers were commonly mentioned throughout all FGD and individual interviews. As with bread, mentions of them were usually qualified “without sugar”. Reasons for eating them fell into the following categories:

Meal substitute:

“When he is supervising his cocoa farm, he doesn’t have time for lunch so he takes biscuits to the farm for lunch.” (53-year-old rural female)

Sustenance between meals:

“Biscuits can sustain her until she takes her next meal. Biscuits are ok and healthy.” (38-year-old rural female)

Convenience:

“We eat cabin biscuits because we get hungry and it’s easy, even though we’ve been asked not to eat it by the doctor.” (73-year-old urban female).

Many people mentioned that biscuits and crackers were fine, or even good, for people with diabetes. Three used the word “healthy” reasoning that they either don’t contain sugar or that they are fat free. One person added that they were healthy because “they are just made of flour.” Only one person mentioned that the doctor advised against eating them. The types mentioned by name were Cabin Biscuits, Cream Crackers, Soda Biscuits, all of which are made with refined wheat flour. In general, participants distinguished sugary foods from bread, crackers and unsweetened biscuits as illustrated well by this quote: “My husband doesn’t take soft drinks but he has biscuits without sugar if there is no bread.” (56-year-old urban female)

#### Portion size

Most participants emphasized the importance of quantity in terms of moderating or reducing food intake. Some used specific language such as “one fistful of banku” and “two fingers of plantain”, though many spoke in general terms such as “everything should be in moderation” and “reduce the quantity”. Some statements, however, suggested confusion about the importance of portion size such as “the size of bread should be the size of a sardine can. If we don’t feel that is enough, then we take some more” (38-year-old urban female caregiver who also has diabetes). Both urban and rural participants mentioned that people with diabetes should eat more soup and stew than starch. Many complained, though, that the recommended quantity of food leaves them feeling unsatisfied. For instance, a 52-year-old urban female stated, “I have stomach aches from the recommended quantity because I don’t get satisfied”.

### Access to fresh produce

Only two urban and one rural participant(s) reported having poor access to markets selling fresh produce, though they could find a limited selection of produce in corner stores. In many cases, there were small markets nearby and larger ones with a more extensive variety of fresh foods a bit farther, but accessible, nonetheless. Seasonal availability was frequently referred to as a limiting factor to year-round access to certain fruits and vegetables.

### Economic factors

Some participants expressed concern about the recommended ratio (more vegetables: less starch) and its impact on household economics. Specifically, they reported that higher vegetable intake would drive-up food expenditures for the whole household as meals are prepared for family members collectively rather than individually. All participants in one FGD agreed with this statement: “If we had the means, we would but it’s too costly. You have to do it for your whole family, not just yourself.” On the other hand, several participants said that it would not be difficult to add more vegetables to the diet if it would improve diabetes-related health outcomes. In one case, a caregiver (38-year-old rural female) even suggested a timesaving measure to facilitate this: “Yes, I could do it. It’s not difficult to prepare more vegetables. It would be convenient for me because I could prepare it in large quantities to keep for the week.” Altogether, more urban (60%) than rural (33%) participants reported cost to be a barrier to increasing vegetable consumption.

A theme regarding the economics of choosing ‘local’ versus ‘exotic’ vegetables emerged in the FGDs. For example, some participants mentioned the high cost of vegetables as a barrier to following the recommendation to increase vegetable consumption. In two FGDs, however, this topic spurred lively discourse on choosing affordable ‘local’ vegetables over their costly ‘exotic’ counterparts. For instance, a 57-year-old urban male asserted “We should cultivate the habit of eating our local vegetables which are equally healthy and not necessarily patronize the exotic if they are too costly.” Another urban participant (55-year-old female) stated that she had actually sought out information about cultivating leafy greens in order to increase her vegetable consumption. In another case, an urban male and urban female both noted that vegetables are expensive but that there are healthy and less costly alternatives among them. Another urban female specifically identified several less expensive local options as jute leaves, cabbage and garden eggs (a type of eggplant).

Of the caregivers interviewed (all female), three rural and two urban, did not find vegetables to be expensive. Each of the remaining caregivers mentioned economizing by choosing inexpensive, local vegetables, as well as in-season vegetables. Cocoyam leaves, garden eggs, tomatoes, squash seeds, kwahunsusua (a leafy green, fruit-bearing, wild plant) and plantains were all examples of lower cost vegetables provided by the caregivers. For example, a 53-year-old rural female was very specific about which vegetables were more economical: “Cabbage, carrots and other exotic vegetables are quite expensive so I don’t normally use them in food preparation. I use more cocoyam leaves, garden eggs, tomatoes, kwahunsusua, and I add squash seeds.” Notably, participants from both groups (56% rural; 44% urban) identified ‘local’ and ‘in season’ vegetables as a cost-effective means to overcoming this barrier.

## Discussion

In this urbanizing region of Ghana, nutritional knowledge, attitudes and practices among T2DM patients and their caregivers are remarkably comparable among both urban and rural dwellers, apart from the perception of the cost of vegetables. The dieticians’ recommendation to avoid added sugar and sweet-tasting foods were understood and followed by all participants and caregivers. This is consistent with an earlier study conducted in Ghana on the social knowledge of diabetes where there was a strong link made between sweetened foods and diabetes [20]. However, this link was not made clearly to fruits, and there was inconsistency in beliefs about the health effects of fruits. It would be beneficial to increase awareness and to develop consistent messaging about fruits’ effect on blood sugar.

There appears to be confusion and misconception concerning other aspects of disease management, as well. Food portioning, for example, was mostly referenced in abstract terms (e.g., “reduce amount” and “in moderation”), or seemed arbitrary to the participants. Further, the dieticians’ recommended portion sizes were generally considered insufficient. Similar results were found in a 2010 study on non-adherence to diabetes dietary regimens among 370 West Africans. In this study, two contributory factors to non-adherence were identified as: difficulty in estimating desired portion sizes and frustration due to the restriction [21]. Therefore, additional time should be spent on helping people understand portion size. Problem solving around satiety may also be helpful in promoting adherence among diabetic patients and caregivers such as wives cooking for their husbands.

There were opposing perspectives among participants regarding the economics of increased vegetable intake, with many advocating the use of “local” vegetables as a means of mitigating cost. A recent study of consumer preference and marketability of Traditional Leafy Vegetables (TLVs) in Kumasi found a high degree of consumption and socio-cultural value for these vegetables in this region [22]. In fact, the authors recommend “intensive education” regarding the nutritional benefits of traditional leafy greens to increase consumption among Ghanaians. A different study conducted in Ghana concluded that the economics of recommended foods in relation to diabetes self-management should be addressed [20]. A targeted campaign to increase TLVs consumption among people with diabetes could be highly beneficial as many people already appreciate the economic value of local vegetables and prefer them, in general.

Regarding plantains, confusion about dietary recommendations persists despite active efforts among the health center staff to dispel misconceptions about them. Currently, both large and small plantain types are recommended on diet plans, with fewer “fingers” recommended for the larger variety. In the health talks, messaging focused on limiting consumption of plantains because of carbohydrate content as well as dispelling a notion that consuming plantains can cure diabetes. Recently, however, a growing number of studies have pointed to the positive health effects of plantains on diabetes, especially when green or roasted [23–25]. A snack of roasted plantains and peanuts happens to be commonly found among street food vendors in Ghana. This snack, known familiarly as ‘Kofi broke man’, is inexpensive and considered a poor person’s food. In developing new dietary guidelines, it would be beneficial to incorporate evidence from recent research on plantains with special attention paid to any stigma related to socio-economic class with regards to messaging

Bread, biscuits and crackers are sold ubiquitously in Ghana and their consumption is common among people with diabetes. Understanding about nutritional relevance of these foods is low, however. In particular, there seems to be a lack of knowledge regarding the relationship between starches and the glycemic index. Therefore, nutritional recommendations should directly address bread, biscuits and crackers, and messaging should be consistent. Diabetes education should include information about the relevance of starches, including refined carbohydrates, to blood sugar levels. Slabber developed a list of higher glycemic index foods with lower glycemic alternatives for use in South Africa [26]. Perhaps a similar list could be developed for the Ghanaian context as one way to address the problem of bread, biscuits and crackers.

The dietary preferences of both urban and rural youth should be of concern to the public health sector of Ghana. All participants in this study agreed that older and younger generations eat differently. Specifically, the general consensus was that the younger generation prefers processed, highly seasoned and sweetened foods to home-cooked, whole foods. A 2011 study [27] in three regions of Ghana, found that less than half of adolescents consumed fresh fruits and vegetables more than four days per week. Based on the conclusion of this study, as well as the older generation’s observations in this study, special intervention efforts should be directed at Ghana’s youth to prevent diet-related NCDs, such as T2DM. To quote a 56-year-old, urban female with no formal schooling, “I think we should all eat healthy. If the young ate that way, they wouldn’t develop the sickness that older people get, like diabetes”.

### Limitations of the study

For our study, participants were recruited from the diabetes clinic of a hospital and thereby receiving some degree of education about diabetes management from clinic staff and physicians. As a result, their views may not represent all persons with diabetes including the undiagnosed as well as those who pursue non-biomedical treatment for the disease (spiritual healers, for example).

In addition, the FGD participants were not segregated by socio-demographic criteria such as age, gender, educational level, ethnicity or residential status (urban or rural). Power dynamics related to these criteria may have skewed participants’ level of engagement during group discussions.

Lastly, participants identified a variety of information channels for receiving diabetes education. Diabetes patients most commonly cited their health care providers as information sources whereas caregivers mentioned one or more of the following: self-initiated research; the patient him- or herself; friends and family; the radio and; less commonly, from health care providers. This study did not examine how information sources relate to diabetes knowledge among participants. To tailor interventions more effectively, future research should examine whether or not the same information is received and understood consistently between the diabetes patients and their caregivers.

## Conclusions

As a result of the dual burden of infectious and non-communicable disease, rapid urbanization and the rising prevalence of T2DM, it is critical to address the food preferences and dietary choices among people with diabetes in the Kumasi-region. This study identified several themes pertaining to nutritional knowledge; attitudes and practices but additional quantitative studies are needed to reveal trends in actual consumption patterns, as proposed by the WHO. Recommendations on improving diet behaviors include: educating about the relationship between commonly available carbohydrates and blood sugar; spending more time on the importance of portion sizes; evidence-based and consistent messaging on plantains; and a list of lower-glycemic alternatives to high-glycemic foods typically available in the region.

Finally, a critical area for further research involves understanding nutritional knowledge, attitudes and practices among the youth and young adults in Ghana. According to the respondents in this study, today’s younger generations are biased toward less healthy, convenience foods. At the same time, the food landscape in Ghana is becoming increasingly affected by the globalization of processed foods. Given the diabetes prevalence increases projected by the WHO, the IDF, and several studies in Ghana, it is imperative to develop an informed prevention campaign targeting dietary preferences among Ghanaian youth in the imminent future.

### Consent (Adult)

“We obtained written informed consent from the patient for the publication of this report and any accompanying images”.
